# Supplementary figures and images for: Larger and Denser: An Optimal Design for Surface Grids of EMG Electrodes to Identify Greater and More Representative Samples of Motor Units
Source: eNeuro. 2023 Sep 11;10(9):ENEURO.0064-23.2023. doi: 10.1523/ENEURO.0064-23.2023 (PMC10500983; doi:10.1523/ENEURO.0064-23.2023)

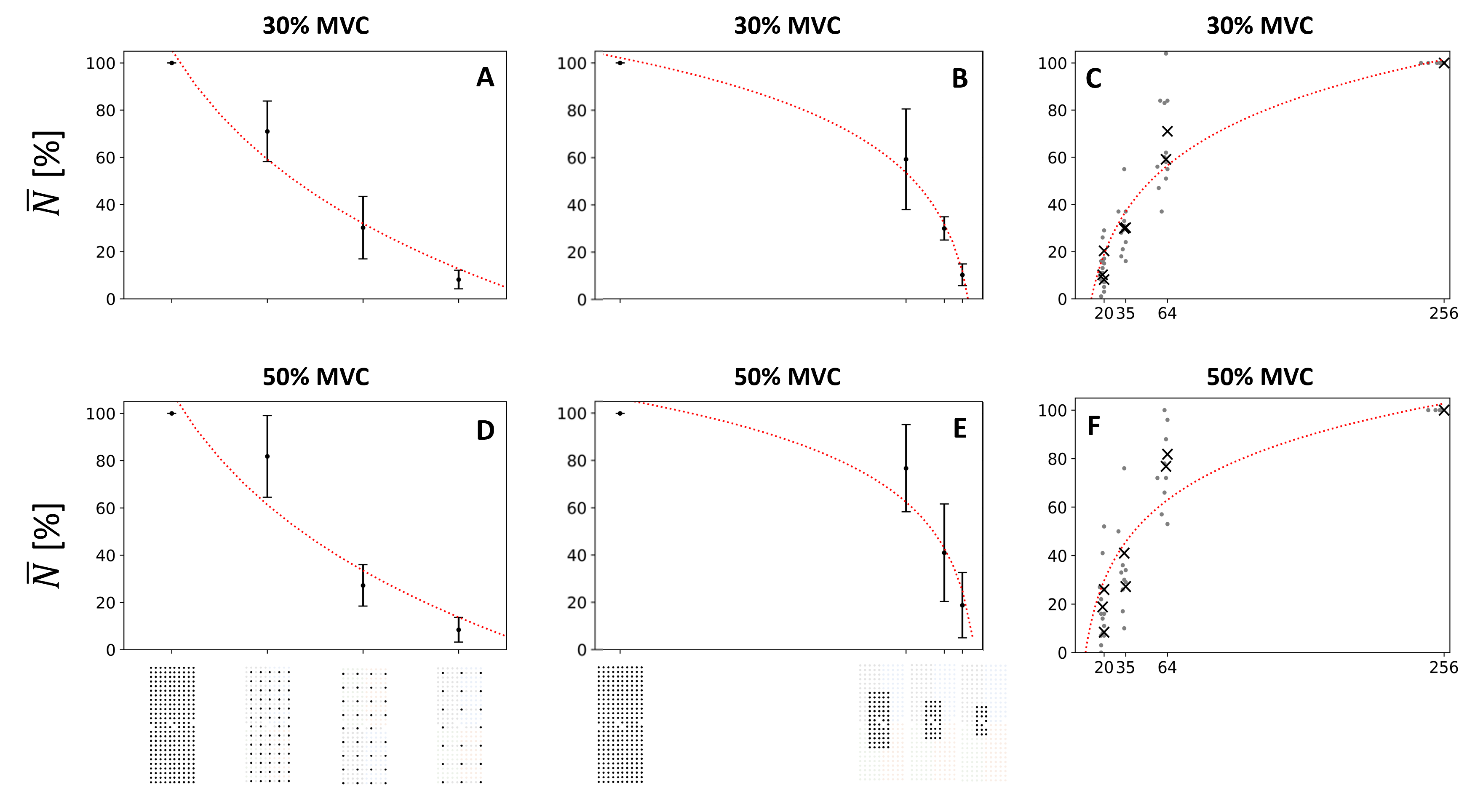

Supplement: Extended Data Figure 4-1 — Effect of the density of the grid (A, D), the size of the grid (B, D), and the number of electrodes (C, F) on the normalized number N¯ of identified motor units at 30% (A–C) and 50% MVC (D–F). N¯ was estimated after decomposing the full grid of 256 electrodes and manually editing the motor unit pulse trains. Vertical bars (A, B, D, E) are the standard deviation of N¯ across subjects, scatters are the individual data points, and crosses are their mean (C, F). Logarithmic trendlines were fitted between the averaged values N¯ and IED, grid size, and number of channels, as in Figures 4-6 of the main document. Here, the trendlines were fitted with the values obtained from the decomposition of the full grid of 256 electrodes. Consistent with the results provided in the main document, N¯ increased with electrode density ( d), grid size (s), and with the number of electrodes (n) following statistically significant logarithmic trendlines (p < 0.05). At 30% MVC, N¯=198−67log⁡(d)(r2=0.92), N¯=−10+31log⁡(s)(r2=0.98), and N¯=−78+32log⁡(n)(r2=0.90). At 50% MVC, N¯=204−69log⁡(d)(r2=0.92), N¯=5+28log⁡(s)(r2=0.98), and N¯=−57+29log⁡(n)(r2=0.90). It is noteworthy that the trendlines exhibited more pronounced plateaus (lower b value in the y=a+b⋅log⁡(x) trendlines) with the decomposition of the full grid of 256 electrodes than with the decomposition of subsets of 64 electrodes. Download Figure 4-1, TIF file. [file enu-eN-NWR-0064-23-s02.tif]

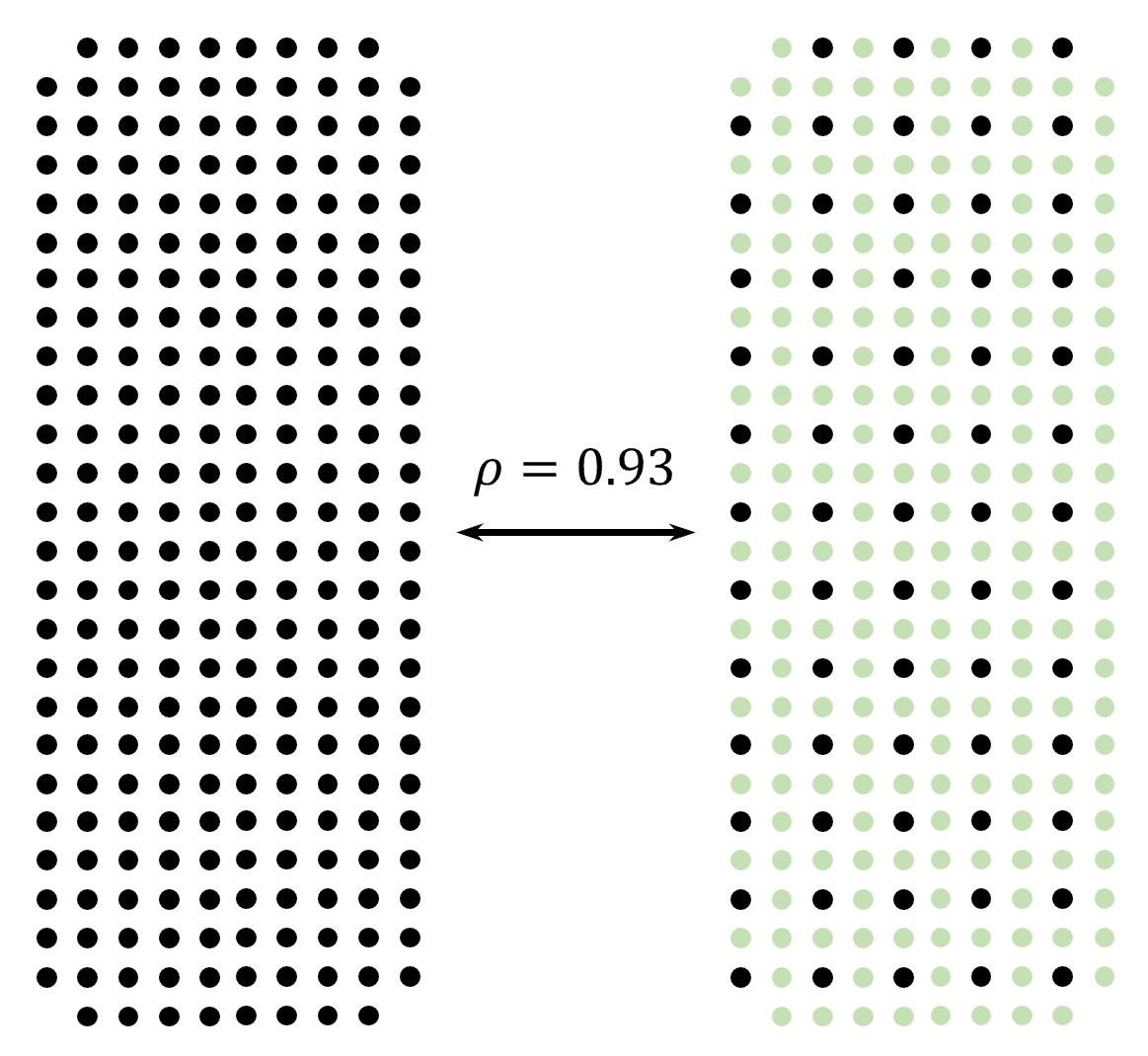

Supplement: Extended Data Figure 4-2 — Correlation ρ between experimentally recorded (left, black) and interpolated (right, green) EMG signals (right, black). Using the ultradense grid of 256 electrodes (2-mm IED) at 30% MVC, we spatially interpolated down-sampled montages of 4 × 9 electrodes with an IED of 8 mm and 5 × 13 electrodes with an IED of 4 mm to generate 5 × 13 (4-mm IED) and 10 × 26 (2-mm IED) grids of electrodes, respectively. In these interpolated grids, 25% of the signals were therefore experimentally recorded (right, black) and 75% interpolated (right, green). After comparing interpolated and experimentally recorded grids of electrodes, we observed that a better signal reconstruction was obtained with the 2-mm IED, with a correlation coefficient of ρ = 0.93 ± 0.09 between recorded and interpolated signals. We identified 4 and 19 motor units from the interpolated grid with a 4- and 2-mm IED, respectively, versus 19 and 24 motor units with the experimentally recorded signals. We only identified the same motor units as identified with the original less dense grids used to generate the interpolation. These results indicate that interpolation is not sufficient to reconstruct signals from a lower spatial sampling. This may be due to the spatial bandwidth which is greater than the inverse of the minimal interelectrode distance used or to the edge effects of the interpolation due to the relatively small size of the grid. Download Figure 4-2, TIF file. [file enu-eN-NWR-0064-23-s03.tif]

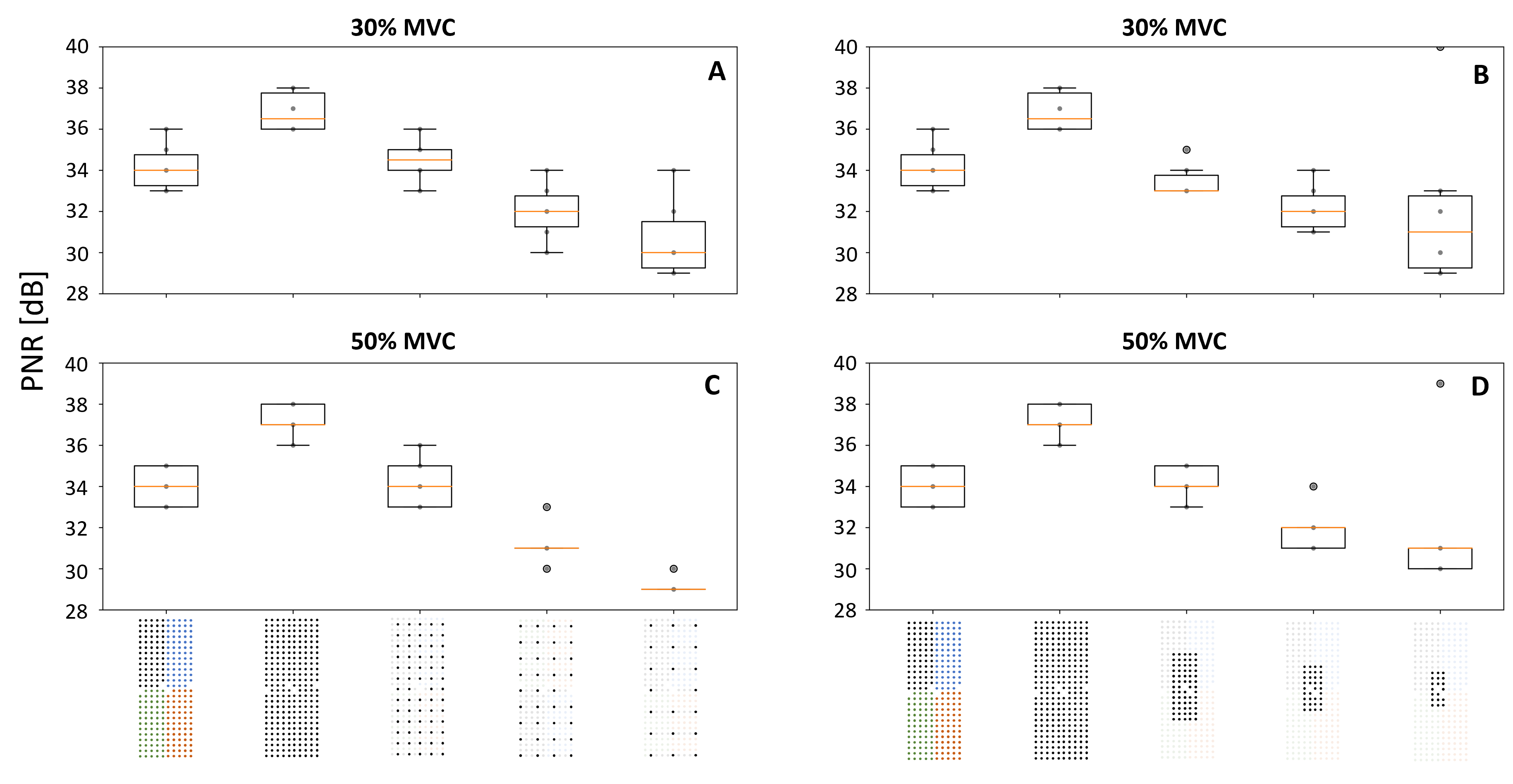

Supplement: Extended Data Figure 4-3 — Effect of the electrode density (A, C) and grid size (B, D) on the average PNR across the identified spike trains at 30% MVC (A, B) and 50% MVC (C, D). The boxplots report the average PNRs per participant (grey dots) and the median (orange line), quartiles, and 95% range across participants. We calculated the average PNR value for the motor unit spike trains (PNR > 28 dB) identified in each subject and condition. The average PNR across identified motor units increased together with both the density and the size of the grid. The lowest PNR values were observed with 16 mm-IED (30 ± 1.8 dB at 30% MVC and 29 ± 1.2 dB at 50% MVC) and with a grid of 2 cm2 (31 ± 0.9 dB at 30% MVC and 30 ± 0.9 dB at 50% MVC). The highest PNR was observed with 4 mm-IED and a grid of 36 cm2 (36 ± 0.7 dB at 30% MVC and 37 ± 0.7 dB at 50% MVC), enabling the operators to quickly edit the identified motor units. Download Figure 4-3, TIF file. [file enu-eN-NWR-0064-23-s04.tif]
